# Matching between maternal knowledge about infant development and care for children under one year old

**DOI:** 10.1590/1518-8345.5967.3675

**Published:** 2022-10-17

**Authors:** Ellen Cristina Gondim, Luiz Guilherme Dácar da Silva Scorzafave, Daniel Domingues dos Santos, Nayara Cristina Pereira Henrique, Fabrícia de Magalhães Pereira, Débora Falleiros de Mello

**Affiliations:** 1 Universidade de São Paulo, Escola de Enfermagem de Ribeirão Preto, PAHO/WHO Collaborating Centre for Nursing Research Development, Ribeirão Preto, SP, Brazil.; 2 Universidade de São Paulo, Faculdade de Economia, Administração e Contabilidade de Ribeirão Preto, Ribeirão Preto, SP, Brazil.

**Keywords:** Child Development, Child Care, Mothers, Knowledge, Mother-Child Relations, Primary Care Nursing, Desenvolvimento Infantil, Cuidado da Criança, Mães, Conhecimento, Relações Mãe-Filho, Enfermagem de Atenção Primária, Desarrollo Infantil, Cuidado del Niño, Madres, Conocimiento, Relaciones Madre-Hijo, Enfermería de Atención Primaria

## Abstract

**Objective:**

to analyze maternal knowledge about infant development and its matching to the care offered to children during their first year of life.

**Method:**

a longitudinal and prospective study, in the stages of pregnancy and of the child’s 12^th^/13^th^ month of life. Interviews were applied to 121 women in a Brazilian city, based on 21 items selected from the Knowledge of Infant Development Inventory, related to the first year of life. Calculation of rates of correct answers was used, as well as regression by Ordinary Least Squares and White’s standard error.

**Results:**

the participants who answered correctly more aspects have more years of study, are older and present high family incomes. When the “having a partner or not” variable was considered, the correct answers presented a discrete fluctuation. Regarding the themes, there were more correct answers to aspects about health, safety and infant development milestones. Primiparous mothers were more likely to wean, overprotect and have children using electronic devices, and less likely to seek information about child care.

**Conclusion:**

there was matching between some maternal knowledge and execution of child care. The connection between them is relevant to indicate in detail the unknowns and uncertainties and to improve positive knowledge, contributing to promoting early childhood development.

## Resumo

**Objetivo:**

analisar os conhecimentos maternos sobre o desenvolvimento infantil e sua correspondência aos cuidados ofertados à criança no primeiro ano de vida.

**Método:**

estudo longitudinal, prospectivo, nas etapas da gestação e 12º/13º mês da criança. Foram aplicadas entrevistas a 121 mulheres de um município brasileiro, baseadas em 21 itens selecionados do Inventário de Conhecimento sobre o Desenvolvimento Infantil, relativos ao primeiro ano de vida. Utilizou-se o cálculo de taxas de acertos, regressão por Mínimos Quadrados Ordinários e erro padrão de White.

**Resultados:**

as participantes que acertaram mais aspectos possuem mais anos de estudo, mais idade e renda familiar elevada. Os acertos quando considerada a variável “ter ou não companheiro/a” apresentaram discreta oscilação. Quanto às temáticas, acertaram mais aspectos sobre saúde, segurança e marcos do desenvolvimento infantil. Mães primíparas apresentaram maior probabilidade de desmame, superproteção e da criança ter contato com aparelhos eletrônicos, e menor probabilidade de buscar informações sobre cuidados infantis.

**Conclusão:**

houve correspondência entre alguns conhecimentos maternos e a execução dos cuidados da criança. A conexão entre eles é relevante para indicar detalhadamente os desconhecimentos e as incertezas e aprimorar os saberes positivos, contribuindo para promover o desenvolvimento na primeira infância.

## Resumen

**Objetivo:**

analizar el conocimiento materno sobre el desarrollo infantil y su correspondencia con el cuidado que se le brinda al niño en el primer año de vida.

**Método:**

estudio longitudinal, prospectivo, en las etapas de gestación y de los 12/13 meses del niño. Se les realizaron entrevistas a 121 mujeres de un municipio brasileño, basadas en 21 ítems seleccionados del Inventario de Conocimiento sobre Desarrollo Infantil, relacionados con el primer año de vida. Se utilizó el cálculo de las tasas de aciertos, la regresión de Mínimos Cuadrados Ordinarios y el error estándar de White.

**Resultados:**

las participantes que tuvieron una mayor cantidad de aciertos tienen más escolaridad, más edad e ingreso familiar alto. Los aciertos vinculados a la variable “tener pareja o no” mostraron una ligera oscilación. En cuanto a los temas, se registraron más aciertos en los ítems sobre salud, seguridad e hitos del desarrollo infantil. Las madres primíparas presentaron mayor probabilidad de destete, sobreprotección y de que el niño estuviera en contacto con dispositivos electrónicos, y menor probabilidad de buscar información sobre el cuidado infantil.

**Conclusión:**

hubo correspondencia entre algunos conocimientos maternos y los cuidados que le brindaron al niño. La conexión entre ellos es importante para indicar detalladamente las incógnitas y las dudas y mejorar el conocimiento positivo, que contribuye a promover el desarrollo infantil temprano.

**Highlights**

(1) Mothers with higher income and schooling levels know more about development.

(2) Mothers answer correctly more questions about health, safety and infant development milestones.

(3) Primiparous women present more weaning, overprotection and children with electronic devices.

(4) Internet searches suggest active consumption of information, indicating vulnerabilities.

(5) The relationship between knowing and practicing care detects child care outcomes.

## Introduction

The first years of life are fundamental and cover important variables for human development, such as the family environment and its characteristics, stimuli, practices and interactions^1^ ^-^ ^2^. Diverse scientific evidence suggests the importance of parental caregivers in everyday life and needs to support them, in order to favor a healthy early childhood and adequate development^3^ ^-^ ^5^. 

Absence of attentive and affective care in early childhood can impair learning and memory, affecting both physical and socio-emotional health^4^ ^-^ ^5^, increasing the chances of unfavorable outcomes for infant development and making it difficult to reach the child’s potential. Parental interventions for children under two years of age in low- and middle-income countries have been identified as valuable, highlighting that more in-depth research studies are necessary, integrating responsive care and environmental risk factors^6^. 

In general, the mother is recognized as the closest parental figure to perform the child’s everyday care, although parental contexts have been increasingly discussed regarding maternal overload, partnership status and gender, socioeconomic tensions related to the provision of child care^7^. The maternal perception about the first years of life is important, and it was found that the mothers’ knowledge is more focused on the physical aspects^8^. 

In the context of infant development, maternal knowledge focuses on motor aspects, with gaps in cognitive, socio-emotional and parental interaction skills with the child^9^. Studies based on the Knowledge of Infant Development Inventory (KIDI), an instrument used to measure knowledge about infant development, found lower schooling levels associated with a lower range of knowledge^10^ and higher scores of knowledge in older mothers, with higher instruction levels and better economic incomes^11^.

In this survey, knowing whether aspects of healthy development are inherent to maternal knowledge about the care provided to children in their first year of life motivated the research, due to the relevance of parental care and of the interactions for good development in the first years of life. Given the above, the relevance of identifying the correlation of socioeconomic variables in this process is enhanced. Thus, the objective was to analyze maternal knowledge about infant development and its matching to the care offered to children during their first year of life.

## Method

### Study type

A longitudinal and prospective study conducted in two stages in a health district from a Brazilian municipality in the inland of São Paulo. 

### Data collection locus

In both stages, the interviews were individual by means of home visits (HVs). 

### Period

The survey of the number of pregnant women occurred with the support from the teams of units with Family Health Strategy (FHS), continuously and respecting the predetermined time frame between November 1^st^, 2017 and December 31^st^, 2018. The purpose was to select the participants who were in the last trimester of pregnancy, considering the premise that the children would complete their 12^th^-13^th^ month of life by January 31^st^, 2020, and the interviews were finished in the same period.

### Sample

A total of 529 participants in their last gestational trimester were identified. In all, 110 did not meet the inclusion criteria, 173 were removed due to the exclusion criteria and 26 refused to participate, resulting in 220 eligible women to comprise the sample. However, 43 participants were excluded due to discontinuity criteria and 56 were considered as losses for not being found to conduct the HVs. 

### Selection criteria

The inclusion criteria were the followings: i) pregnant women in the last trimester of pregnancy, of usual risk, over 18 years of age; and ii) registered and undergoing follow-up in a unit with FHS, in the area covered by the health district. The exclusion criteria were as follows: i) risk pregnancies, ii) women deprived of their freedom or hospitalized, and iii) not speaking Portuguese. Regarding the discontinuity criteria, the following were established: i) change in the coverage area of the health district in question, and ii) mothers’ decision to withdraw their participation. Those women who were not found after three attempts to conduct the HV were considered as losses. 

### Participants

Pregnant women/mothers were the central participants of the study, with data collection with the same participant in the last trimester of pregnancy (pregnant woman) and between the 12^th^ and 13^th^ month after birth of the child (mother). They accounted for a total of 121 participants.

### Study variables

Dependent variable: maternal knowledge. The independent variables were linked to the care offered to the child, for the identification of possible correlations between maternal knowledge and the execution of care, related to the following: occurrence of weaning, child’s age at weaning and introduction of new food options, interaction/play of the mother with the child during care, positive interaction of the child with the mother, search for information sources about infant development, use of electronic devices by children, habit of reading to the child, stimuli to correct verbalization, overprotective maternal behavior, and type of care provided to the child. Variables related to characterization: maternal age group, schooling, income and marital status.

### Instruments

The Knowledge of Infant Development Inventory (KIDI) was used [its translated and adapted version into Portuguese known as *Inventário de Conhecimento sobre o Desenvolvimento Infantil* (ICDI)]^12^.

KIDI has 75 questions that address aspects going from birth to 6 years old age. In the current research, 21 questions from KIDI were selected, related to the child’ first year of life. In each of the 21 questions, the participants had the option of answering “I agree”, “I disagree” or “I’m not sure”, the last option when the participant mentioned indecision and/or not knowing how to choose the “I agree” or “I disagree” statement. Assignment of correct of incorrect answers refers only to the “I agree” and “I disagree” options.

KIDI organizes its questions in four domains. The Parental Practices domain covered 7 questions in this study, with elements of the parental caregivers’ behavior and actions, for example: “the baby should not be carried on the lap when being fed because in this way it will want to have lap all the time”, or “talk to the baby about things it is doing helps in its development”. The Infant Development Norms and Milestones domain is related to parental knowledge about probable periods of skills acquisition by the child and, in this research, it was explored by a single element: “babies do some things only to cause problems for their mother or father, such as crying for a long time or getting their diapers dirty”, considered relevant for the age group studied. The Principles domain included 10 questions in this research, focused on the notions of the child’s development process and general skills, for example, “babies understand only the words they can speak”, or “a little sister or brother can start peeing in bed or sucking the finger when a new baby arrives in the family”. The Health and Safety domain was addressed in 3 questions, involving solid food intake by children under one year old such as popcorn, use of pillows in the crib, and not offering solid food when the child shows changes in bowel elimination.

### Data collection

In the pregnancy stage, a structured interview was conducted with application of a questionnaire on sociodemographic, economic and obstetric aspects to characterize the participants, as well as topics on infant development, based on KIDI items. 

In the second stage, between the child’s 12^th^ and 13^th^ month of life, a questionnaire was applied regarding the infant’s profile, care and infant development, with questions related to the aspects of the first stage. The intention was to identify if the care provided during the child’s first year of life is correlated to the maternal knowledge about pregnancy. The type of care chosen (locus/person) for the child was also identified between the 12^th^ and 13^th^ months of life. A question was also added about the maternal habit of reading books to the child, in view of the contribution to the cognitive and language spheres, as well as about the information sources sought by the mothers as resources for deepening on the theme of infant development. 

Each of the stages, conducted through HVs, lasted a mean of 40 minutes.

### Data treatment and analysis

In the descriptive statistics analysis, the relative frequency of the variables investigated was calculated. The “maternal knowledge” variable was considered as dependent and the others, as independent. Calculation of the rates of correct answers on infant development was used, as well as regression by Ordinary Least Squares (OLS) and White’s robust standard error. 

### Ethical aspects

The research was authorized by the Research Project Evaluation Committee of the Municipal Health Department of the aforementioned municipality and approved by the Research Ethics Committee (CAAE No. 70838817.2.0000.5393). A Free and Informed Consent Form was used in two original copies, which were read and signed, one being delivered to the participant, explaining the research objective, guarantee of anonymity and autonomy for withdrawal at any stage of the study, without prejudice or harms.

## Results

The participants’ profile is mostly comprised by the age groups between 18 and 25 years old (43.8%) and between 26 and 35 years old (40.5%). Regarding self-declared skin color, a large percentage consists of black-/brown-skinned (52.1%) and white-skinned (43.8%) women, with Complete High School (50.4%). Most of the participants live with a partner (80.2%). In relation to the number of people living in the household, most of them (72.7%) live with up to three individuals, and the rest of the participants live with up to 14 people.

Regarding occupation, a large percentage works outside the house (48.8%) and others are housewives (29.7%). Some were unemployed (19.9%), none of them stated being students and some preferred not to answer (1.6%). The predominant family income corresponds to less than three minimum wages (47.1%), followed by the range from three to less than five minimum wages (28.9%). Of all the participants, 18.2% receive governmental assistance. 

It was identified that 38.0% were in their first pregnancy, 25.6% in the second, 18.2% in the third, and another 18.2% in their fourth pregnancy or more. 52 (43.0%) of the participants were primiparous. Regarding number of children, it varied from one to seven live children. Most of the births were through normal deliveries (61.2%), with gestational ages between 37 and 41 weeks (87.6%) and birth weight between 2,500 g and 3,999 g (90.9%). 

Table 1 shows the participants’ correct answers in the infant development domains.

**Table 1:** Distribution of the pregnant women’s correct answers in each domain of the Knowledge of Infant Development Inventory. Ribeirão Preto, SP, Brazil, 2018-2020

Domains	Rate of correct answers (%) N=121	
Parental Practices	85	69.4
Principles	64	52.8
Health/Safety	87	72.4
ID Norms/Milestones	104	85.9

Considering the group of participants and the compilation of all 21 questions addressed, the mean of correct answers is 62.9%. As for the number of questions, the correct answers were more expressive in the range from 11 to 15 questions (61.2%) and less expressive from 16 to 21 questions (20.6%) and from 1 to 10 questions (18.2%). 

Table 2 presents the percentage of correct answers about infant development.

**Table 2:** Distribution of the percentage of pregnant women according to the correct answers given to questions about infant development, considering KIDI and the variables under study. Ribeirão Preto, SP, Brazil, 2018-2020

Variable	Rate of correct answers (%)			
	From 1 to 5 questions	From 6 to 10 questions	From 11 to 15 questions	From 16 to 21 questions
**Age group**				
18-25 years old (n=53)	1.9	18.9	60.4	18.9
26-35 years old (n=49)	0.0	12.2	67.3	20.4
≥36 years old (n=19)	5.3	21.0	47.4	26.3
**Schooling**				
Incomplete Elementary School (n=15)	6.7	20.0	73.0	0.0
Complete Elementary School (n=35)	0.0	22.8	65.7	11.4
High School (n=61)	1.6	14.7	54.1	29.5
Higher Education (n=10)	0.0	0.0	70.0	30.0
**Marital status**				
With a partner (n=97)	2.1	17.5	58.8	21.6
Without a partner (n=24)	0.0	12.5	70.8	16.7
Income*				
Up to 1 minimum wage (n=11)	18.2	63.6	9.1	9.1
From 1 to <3 minimum wages (n=57)	22.8	56.1	21.0	0.0
From 3 to <5 minimum wages (n=35)	8.6	68.6	22.7	0.0
From 5 to 15 minimum wages (n=11)	9.1	54.5	36.4	0.0
Not reported (n=7)	14.3	71.4	0.0	14.3

*Minimum wage in force = R$ 1,045.00, Brazil, 2020^13^.

In terms of income, the highest frequencies of correct answers are around 6 to 10 KIDI questions.

Regarding the KIDI questions, they are stratified by domains; the participants presented higher frequency of knowledge in the Health/Safety (72.4%) and Infant Development Norms/Milestones (85.9%) domains.

In the analysis of the results, the means of correct answers from the compilation of the 21 questions applied and of the KIDI domains were also considered, performing in both crossing of the diverse information with the variables of interest pointed out.

In terms of the age group, it was identified that the mean of correct answers was 63.0%. Pregnant women between 18 and 25 years old scored 60.6%, between 26 and 35 years old, 65.1%; and those aged 36 years old or more scored 63.4%, considering all 21 questions. When the inventory domains are verified, the rates of correct answers are higher for Health/Safety and Infant Development Norms/Milestones, and those aged between 26 and 35 years old also gave correct answers in the Parental Practices domain frequently. 

Regarding schooling, it was verified that the more years of study, the higher the rates of correct answers, although the number of participants in each category is different. Incomplete Elementary School, Complete Elementary School and High School obtained 53.6%, 60.3% and 65.1% of correct answers, respectively. The participants with Higher Education answered correctly 72.4% of the questions. The Infant Development Norms/Milestones domain presented higher rates of correct answers in all schooling levels, with increasing rates according to more years of study. The second domain with the highest rate of correct answers for participants with Elementary School is the Health/Safety domain (Complete: 80.0%; Incomplete: 73.3%), while for those with High School and Higher Education, the percentages in the Parental Practices domain were 71.2% and 81.4%, respectively. 

A discrete variation in the rate of correct answers was noticed when comparing presence of a partner (62.9%) to no partner (62.7%), whether it is the child’s father or not. The highest frequencies of correct answers were in the Health/Safety (72.5%) and Infant Development Norms/Milestones (84.5%) domains for the participants that have a partner. In the absence of a partner, there were also more correct answers in the Health/Safety (72.2%) and Infant Development Norms/Milestones (91.7%) domains, including a slight increase in such rates for the Norms/Milestones domain, when compared to those who have some type of relationship. 

The participants’ income profile ranged from earning less than one minimum wage, with the help of the *Bolsa Família* program, to monthly family incomes of up to 15 minimum wages. The number of participants by category varied and the mean of correct answers did not differ among them. In the set of participants in relation to income, the rate of correct answers was not less than half of the questions, although it is noted that those with higher incomes tend to answer more questions correctly. 

Given the above, it is noted that maternal knowledge about infant development for the questions proposed by KIDI is often focused on the aspects of health and infant development milestones, even when associated with other variables of interest such as age, schooling, income and presence of a partner. 

Thus, the participants showed that it is not appropriate to offer solid food such as peanuts or popcorn to nine-month-old children, they point out that placing a soft pillow in the crib is not a good and safe way to help the child sleep better, or that it is not necessary to stop feeding a child under one year old with solid food when it has diarrhea, aspects that encompass the Health/Safety domain. In addition, the participants pointed out that the infants do not indulge in certain behaviors, such as crying for a long time, in order to cause problems to the parental caregivers. 

On the other hand, the participants showed lack of knowledge in the Development Principles and Parental Practices domains. There was unawareness or doubt regarding the principles involving children understanding not only the words they can speak, or not learning everything in their language by copying what they hear from adults; the child’s individuality not being formed at six months of age; and the fact that some children do not like being on their mother’s lap. In the Parental Practices, uncertainties and unknowns surround aspects related to the fact that the mother does not really get involved with her baby until it starts to smile and look at her; as well as to comforting the child, holding it and talking to it while crying is not an act that will “spoil” the child; or that when disciplining the child in a day for inappropriate behavior, it should be guided again if it repeats it, not being something that depends on the maternal mood on the day in question. 

At the children’s 12^th^-13^th^ month of life, the participants answered about practices and types of care (locus/person) offered to the child, presented in Table 3.

**Table 3:** Distribution of the percentage of the variables referring to child care between the 12^th^ and 13^th^ month of life, according to the age group of the mothers from a health district of a Brazilian municipality. Ribeirão Preto, SP, Brazil, 2018-2020

Child care practices	(n=121)%	Maternal age group (%)		
		18-25	26-35	≥36
**Weaning at the first year of life**				
Yes	41.3%	42.0%	42.0%	16.0%
No	58.7%	45.1%	39.4%	15.4%
**Child’s age in the cases where there was weaning**				
≤6 months old	27.3%	51.5%	36.4%	12.1%
7-11 months old	13.2%	25.0%	50.0%	25.0%
≥12 months old	0.8%	0.0%	100%	0.0%
**Child’s age when other food products were introduced**				
<6 months old	96.7%	44.4%	39.3%	12.1%
≥6 months old	3.3%	25.0%	75.0%	0.0%
**Interaction/Games between the mother and the child during the care provided**				
Yes	99.2%	43.3%	40.8%	15.8%
No	0.8%	100%	0.0%	0.0%
**Child’s positive interaction when the mother talks/interacts**				
Yes	100%	43.8%	40.5%	15.7%
No	0.0%	0.0%	0.0%	0.0%
**Information sources sought by the mothers about child care**				
None	25.6%	29.0%	51.6%	19.3%
Social networks	28.1%	52.9%	32.3%	14.7%
Blogs and websites	40.5%	42.7%	40.8%	16.3%
Others	5.8%	71.4%	28.6%	0.0%
**The child uses electronic devices offered by the mot**her				
Yes	79.3%	46.9%	35.4%	17.7%
No	20.7%	32.0%	60.0%	8.0%
**The mother has the habit of reading books to her child**				
Yes	27.3%	42.4%	45.4%	12.1%
No	72.7%	44.3%	38.6%	17.0%
**Maternal stimuli for the child to say the correct names of objects**				
Yes	93.4%	43.7%	40.7%	15.9%
No	6.6%	50.0%	37.5%	12.5%
**Overprotecting maternal behavior**				
Yes	81.8%	45.4%	39.4%	15.1%
No	18.2%	36.7%	45.4%	18.2%

Regarding interaction, the participants mostly reported talking and interacting with their children, in addition to stimulating correct verbalization of object names by the child.

The child care elements obtained through the mothers’ answers in the child’s 12^th^ and 13^th^ month of life were interconnected with the KIDI domains, suggesting correspondence with the domains proposed by the inventory. Thus, the positive interaction and the stimulus to correct verbalization, relevant points in early childhood, were presented as care actions by the participants and suggest coherence with the Infant Development Norms/Milestones domain, which displayed a higher rate of correct answers during pregnancy, despite having a single question in this domain. 

Weaning occurred in 41.3% of the children, most frequently during the first six months of life, introducing other food products before this age. The introduction of other food options in the child’s dietary routine is linked to the Health/Safety domain, which presented a higher rate of correct answers when the participants answered during their pregnancies. 

Maternal interaction with the child, overprotective behavior and search for information about infant development can be linked to the Parental Practices domain. During pregnancy, the participants presented unknowns and uncertainties in the Parental Practices domain, with lower rates of correct answers when compared to other domains proposed by KIDI. In the 12^th^-13^th^ month of life there were significant answers in the care measures related to interacting with the child and indulging in overprotective behaviors, in addition to seeking information about infant development more frequently on online platforms.

The maternal habits of reading for and with the child and offering electronic devices and the type of child care (locus/person) can be linked to the domain of Principles on infant development. From the perspective of the care chosen, the majority (47.1%) decided not to work outside to stay with the child, followed by those who leave the children at home under the care of other relatives or of a daycare mother (19.0%). The third care option most chosen is resorting to daycare centers (18.2%), followed by other care modalities (6.6%), such as taking the child to the workplace. Private daycare centers appear as the last option (6.6%). 

To analyze matching between maternal knowledge about infant development and the care offered to the child in the first year of life, a regression by Ordinary Least Squares (OLS) was performed, using White’s robust standard error, adopting a 5% significance level (p-value ≤ 0.05). Table 4 shows the results of the analysis performed.

**Table 4:** Regression analysis by Ordinary Least Squares. Ribeirão Preto, SP, Brazil, 2018-2020

Variable	Age when other food products were introduced	There was weaning	Electronic devices	Overprotecting mother	Reading books	Searching information about care
Age	0.01084	0.00003	0.00672	0.00209	-0.00289	0.27633
	[0.012]	[0.005]	[0.004]	[0.004]	[0.005]	[0.223]
Completed 8^th^ grade	0.58922	0.05114	0.11652	0.00483	-0.11049	-0.00057
	[0.543]	[0.194]	[0.157]	[0.079]	[0.152]	[0.005]
Completed High School	0.61142	-0.16332	-0.05091	-0.20443^*^	-0.16543	0.31686
	[0.561]	[0.177]	[0.155]	[0.093]	[0.15]	[0.181]
Completed Higher Education	1.31888^*^	-0.30591	0.28924	-0.29034	0.01354	0.41102^*^
	[0.562]	[0.21]	[0.147]	[0.161]	[0.233]	[0.162]
No partner	0.16905	0.21762	0.07218	-0.08981	-0.19679^*^	0.63021^*^
	[0.242]	[0.114]	[0.089]	[0.089]	[0.097]	[0.163]
Primiparous	0.89451	-0.66318^*^	0.35043^*^	-0.35813	-0.20247	-0.06497
	[0.543]	[0.169]	[0.167]	[0.336]	[0.169]	[0.095]
Rate of correct answers in KIDI^†^	-0.81439	-0.20117	-0.39013	-0.31886	0.47563	0.68637^*^
	[0.766]	[0.356]	[0.27]	[0.264]	[0.344]	[0,155]
Private daycare center	-0.06752	0.11258	0.15006^*^	-0.35977^*^	0.38533^*^	0.1034
	[0.349]	[0.181]	[0.058]	[0.142]	[0.169]	[0.3]
Public daycare center	-0.63012	0.22502	-0.11608	-0.14535	0.0228	0.07568
	[0.323]	[0.118]	[0.098]	[0.109]	[0.104]	[0.142]
House with relatives or daycare mother	-0.05217	0.03195	-0.04662	-0.0155	0.15255	0.04952
	[0.243]	[0.117]	[0.1]	[0.085]	[0.113]	[0.093]
Completed 8^th^ grade x Primiparous	-1.35396	0.33958	-0.28094	0.44436	0.38431	-0.08212
	[0.684]	[0.236]	[0.197]	[0.34]	[0.222]	[0.104]
Completed High School x Primiparous	-0.56786	0.7487^*^	-0.0905	0.46035	0.35968	-0.47163^*^
	[0.632]	[0.206]	[0.189]	[0.355]	[0.203]	[0.218]
Completed Higher Education x Primiparous	-2.56765^*^	1.12552^*^	-0.41088^*^	0.92251^*^	0.23416	-0.4909^*^
	[0,789]	[0,283]	[0,174]	[0.37]	[0,267]	[0,175]

Note: Standard error in between square brackets. For the care situation *dummies*, the comparison is “She does not work to be with the child”; for schooling it is “She did not complete 8th grade” and for parity it is “Being primiparous”. White’s robust standard error and p-value < 0.05* were used; ^†^Knowledge of Infant Development Inventory.

In the OLS analysis, the marginal effect under the variable was considered. In the case of participants who do not have Higher Education and are multiparous, the child’s age increases from the perspective of introducing other food products by 1.318 months in relation to those who have not completed Elementary School and are not primiparous. In turn, for those who completed Higher Education, being primiparous reduced by 1.249 months the age when other food products were introduced. 

At weaning, those who completed High School and Higher Education, the fact of being primiparous increases the probability of weaning by 8.5% and 54.8%, respectively, in relation to those who did not complete Elementary School. 

The participants with Complete Higher Education have a 6.0% probability of their children using electronic devices. This is faced with the analysis of the marginal effect in relation to those who did not complete Elementary School and to the primiparous women. 

As for the type of care, children in private daycare centers are 15.0% more likely to have contact with electronic devices than those whose mothers do not work outside. 

As for overprotection, children who are in private daycare have the probability of the mother being overprotective reduced by 35.9%, in relation to children whose mothers do not work outside. Mothers with complete High School have the probability of overprotective behavior reduced by 20.4% when compared to those who did not complete Elementary School. In turn, the mothers that have completed Higher Education and are primiparous tend to increase by 92.2% the probability of being overprotective, in relation to those who do not work and are multiparous. 

The fact that the mother does not have a partner reduces by 19.7% the chances that she reads books to her child, when compared to those who do have a partner. The children who are in a daycare center have 38.5% more chances for their mothers to read books to them, when compared to the children whose mothers do not work.

Regarding the search for information, not having a partner increases by 63.0% the probability of the mother seeking information about care, in relation to those who have a partner. On the other hand, if the mother has completed High School and is primiparous, the probability of seeking information about care decreases by 63.0%, in relation to those who have not completed Elementary School and are multiparous. 

In Higher Education, the fact of being primiparous reduces by 8.0% the probability of seeking information about care, in relation to those who have not completed Elementary School and are multiparous. Having Higher Education and being multiparous increase by 41.1% the probability of seeking information about care, in relation to those who did not complete Elementary School. 

Regarding KIDI, a 1% increase in the rate of correct answers increases by 68.6% the chances that the mother will seeks information about care.

## Discussion

In this research, considering the KIDI elements by rates of correct answers, the participants get more correct aspects when they have more years of study, are older and earn high family incomes. When presence of a partner or not as support network was analyzed, the rates of correct answers about infant development presented a discrete fluctuation. Regarding the themes, there were more correct answers regarding the aspects about health, safety and infant development milestones. Among the primiparous mothers, there was a higher probability of weaning, overprotection and of the child having contact with electronic devices, and a lower probability of seeking information about child care.

A number of studies based on the KIDI instrument identified the influence of variables for maternal knowledge of infant development, centered on older mothers with higher schooling, income and occupational status^11^ ^,^ ^14^, similarly to the results of this research, as the participants tend to err less the higher their schooling level and age group. 

The correlation between maternal schooling and rates of correct answers in KIDI^15^ suggests that more years of study are an indicator for greater ability to understand infant development. A number of studies highlight that the more educated mothers are, the greater is the tendency to seek information about parental skills^16^, as well as that mature mothers explore information about parenting more seriously than younger mothers and are more likely to have friends with babies, making it possible to learn from them^17^. It was also studied that women who achieve more formal education tend to have children with more opportunities by inserting them in early childhood education, as a way of transmitting educational advantages through the generations^18^.

In relation to the KIDI domains, other research studies^19^ ^-^ ^20^ indicate that the maternal figure had more correct answers in the Health/Safety domain, corroborating the findings of this survey, and the Infant Development Norms/Milestones domain was pointed out with greater frequency of errors. In another study, the domain with the most correct answers was Infant Development Norms/Milestones, followed by Principles, with a positive correlation between correct answers and maternal schooling^15^. A study^21^ on development stages identified limited knowledge concerning the Parental Practices domain, followed by the domain of the Principles of development. 

Confidence in maternal knowledge itself is discussed^22^, inquiring whether knowledge acquisition is linked to informal knowledge, coming from parental experiences or from the offer in formal education.

In the current research, in relation to the search for knowledge about infant development, the most explored information sources were via the Internet. A study emphasizes that the sources refer to whom mothers turn for information, being pointed out that, first and most often, they access family and friends, complementing the search with personal social network advice and formal sources of health professionals and programs^17^. 

A study pointed out that parental knowledge has a significant association in the child’s cognitive, motor, socio-emotional and language dimensions from the perspective of developmental achievements^23^. Family contexts with higher incomes seek more information and present better levels regarding stimulation of the children, such as playing, telling stories and reading books together^24^. A review study^25^ reinforces that positive interactions between mothers and term infants showed beneficial effects, including sleep organization, temperature and heart rate regulation, improvement of crying and cramps, socio-emotional development, speech opportunities and quality of attachment. In the first year of life, the children that live with mothers that have communicative skills obtain higher verbalization scores at 36 months old^26^. 

Children exposed to positive emotional expressiveness at home present higher socio-emotional competence levels than those whose parental caregivers avoid focusing on emotional experiences^27^. Another aspect is the socialization style of the maternal emotions related to the variation in the child’s empathy^28^. 

The following drew the attention in this research: primiparous mothers who presented increased likelihood of weaning and lower child’s age when introducing other food products were overprotective, made electronic devices available to their children and a reduced likelihood of seeking information about child care. Another fact refers to children in private daycare centers, with an increased probability of using electronic devices when compared to those whose mothers do not work outside the house, reduced probability of overprotection and greater probability of contact with children’s books. The fact that the mother does not have a partner reduces the probability of reading books to the child and increases the chances of seeking information, in relation to those who have a partner. Internet searches suggest active consumption of information and raise concerns about vulnerabilities about false content that amplify unknowns and uncertainties, constituting care challenges for the quality and veracity of the diverse information consulted. Such results are relevant to improving the clinical practice in health care and infant development, taking into account that, increasingly, it is fundamental for adults to understand that brain development is shaped in early childhood and that parenting requires an increase in knowledge^17^.

The relevance of parental knowledge implies identifying more details about its contexts and vulnerability situations, whether they are emotionally more exhausted, distracted and less attentive, or coherent and sensitive to their children^5^. Such nuances can be addressed in home programs by strategies developed by health professionals, for satisfactory responses, especially in more disadvantaged areas^29^ ^-^ ^30^.

The interface between poverty and infant development is extremely relevant. A study found that one in ten children belonging to low-income families has deprivation related to life (lack of items to live) and to the child itself (for example, lack of children’s books)^31^, showing a connection with deprivation for full development. Circumstances vulnerable to infant development, including poverty, low maternal schooling and child abuse, represent major challenges in the face of socioeconomic disparities in several countries^32^. The prevalence of suspected developmental delay and inequalities in early childhood in low- and middle-income countries requires a fruitful movement to meet sustainable development goals, requiring inclusive, equitable and good quality learning opportunities for all^33^.

Parental interventions already evaluated display fidelity to Primary Health Care, with positive impacts on mental, emotional and behavioral health outcomes for parental caregivers and children^5^ ^,^ ^32^. Interventions in this field point to the importance of parenting preparedness from pregnancy, as well as the promotion of infant development with investments in the training of health professionals^34^. 

As for the limitations of this study, the identification of knowledge about infant development centered on the mother figure and the first year of life of the child is pointed put, suggesting an expansion to different parental caregivers and at different early childhood moments in future studies.

## Conclusion

There was matching between some maternal knowledge and execution of child care in the first year of life. The connection between them is relevant to indicate in detail the unknowns and uncertainties and to improve positive knowledge, contributing to promoting early infant development. 

The approximation between parental knowledge and experiences and the care effectively provided is extremely important to monitor gaps in understanding and recognize the demands of the skills development process for and of the child, as well as the aspects regarding health and safety in childhood. 

There is emphasis on the need to increase health professionals’ performance and early childhood education for parental support, based on diverse scientific evidence and on actions that minimize the unknowns and uncertainties about integral development in early childhood, in order to suppress fake news and reduce harms to children and families.
